# Retraction: Epitopes screening and vaccine molecular design of SADS-CoV based on immunoinformatics

**DOI:** 10.3389/fvets.2023.1342203

**Published:** 2023-11-29

**Authors:** Frontiers Editorial Office

Following publication, the editorial office was contacted by the first author regarding the omission of co-authors from the article, due to a misunderstanding of their contributions. All authors of the manuscript have agreed to and requested to retract the published article.

This retraction was approved by the Chief Editors of Frontiers in Veterinary Science and the Chief Executive Editor of Frontiers.

